# Perioperative chemotherapy in colorectal cancer with peritoneal metastases: A global propensity score matched study

**DOI:** 10.1016/j.eclinm.2022.101746

**Published:** 2022-11-24

**Authors:** Peter H. Cashin, Jesus Esquivel, Stein G. Larsen, Winston Liauw, Nayef A. Alzahrani, David L. Morris, Vahan Kepenekian, Isabelle Sourrouille, Frédéric Dumont, Jean-Jacques Tuech, Cécilia Ceribelli, Beranger Doussot, Olivia Sgarbura, Francois Quenet, Olivier Glehen, Oliver M. Fisher, Peritoneal Surface Oncology Group International (PSOGI), Nordic Peritoneal Oncology Group (NPOG), American Society for Peritoneal Surface Malignancy (ASPSM), BIG-RENAPE Groups

**Affiliations:** a Department of Surgical Sciences, Section of Surgery, Uppsala University, Akademiska Sjukhuset, Uppsala 75185, Sweden; b Division of Surgical Oncology, Beebe Healthcare, Lewes, DE, United States of America; c Section of Surgical Oncology, Department of Gastroenterological Surgery, Oslo University Hospital, Sognsvannsveien 20, Oslo 0372, Norway; d St George & Sutherland Clinical School, UNSW Australia, Sydney, Australia; e Department of Medical Oncology, St George Hospital, Sydney, Australia; f Department of Surgery, St George Hospital, Sydney, Australia; g Hôspital Lyon Sud, Hospices Civils de Lyon, Lyon, France; h CICLY, Université Lyon 1, Lyon, France; i Department of Surgery, Institut Gustave Roussy, Villejuif, France; j Department of Oncological Surgery, Institut de Cancérologie de l’Ouest, St Herblain, France; k Department of Digestive Surgery, Centre Hospitalo-Universitaire de Rouen, Rouen, France; l Department of Surgery, Centre Hospitalo-Universitaire de l’Archet II, Nice, France; m Department of Digestive Surgery, Centre Hospitalo Universitaire Dijon Bourgogne, Dijon, France; n Department of Surgical Oncology, Cancer Institute of Montpellier, University of Montpellier, Montpellier, France; o Notre Dame University School of Medicine, Sydney, Australia

**Keywords:** Colorectal cancer, Peritoneal metastases, Cytoreductive surgery, Hyperthermic intraperitoneal chemotherapy, Neoadjuvant chemotherapy, Adjuvant chemotherapy

## Abstract

**Background:**

There is a paucity of studies evaluating perioperative systemic chemotherapy in conjunction with cytoreductive surgery (CRS) and hyperthermic intraperitoneal chemotherapy (HIPEC) in patients with colorectal cancer peritoneal metastases (CRCPM). The aim was to evaluate neoadjuvant and/or adjuvant systemic therapy in CRCPM.

**Methods:**

Patients with CRCPM from 39 treatment centres globally from January 1, 1991, to December 31, 2018, who underwent CRS+HIPEC were identified and stratified according to neoadjuvant/adjuvant use. Crude data analysis, propensity score matching (PSM) and Cox-proportional hazard modelling was performed.

**Findings:**

Of 2093 patients, 1613 were included in neoadjuvant crude evaluation with 708 in the PSM cohort (354 patients/arm). In the adjuvant evaluation, 1176 patients were included in the crude cohort with 778 in the PSM cohort (389 patients/arm). The median overall survival (OS) in the PSM cohort receiving no neoadjuvant vs neoadjuvant therapy was 37.0 months (95% CI: 32.6–42.7) vs 34.7 months (95% CI: 31.2–38.8, HR 1.08 95% CI: 0.88–1.32, p = 0.46). The median OS in the PSM cohort receiving no adjuvant therapy vs adjuvant therapy was 37.0 months (95% CI: 32.9–41.8) vs 45.7 months (95% CI: 38.8–56.2, HR 0.79 95% CI: 0.64–0.97, p = 0.022). Recurrence-free survival did not differ in the neoadjuvant evaluation but differed in the adjuvant evaluation – HR 1.04 (95% CI: 0.87–1.25, p = 0.66) and 0.83 (95% CI: 0.70–0.98, p = 0.03), respectively. Multivariable Cox-proportional hazard modelling in the crude cohorts showed hazard ratio 1.08 (95% CI: 0.92–1.26, p = 0.37) for administering neoadjuvant therapy and 0.86 (95% CI: 0.72–1.03, p = 0.095) for administering adjuvant therapy.

**Interpretation:**

Neoadjuvant therapy did not confer a benefit to patients undergoing CRS+HIPEC for CRCPM, whereas adjuvant therapy was associated with a benefit in this retrospective setting.

**Funding:**

None.

**Research in context**

**Evidence before this study**

PubMed/MEDLINE, EMBASE, and Cochrane were systematically searched from October 10, 2022 with neoadjuvant studies including patients after January 1, 2000 by using MESH words: colorectal, peritoneal metastases, CRS [cytoreductive surgery], HIPEC [hyperthermic intraperitoneal chemotherapy], neoadjuvant chemotherapy. Nine studies were identified with most demonstrating no benefit; however, sample sizes have been too small to evaluate this appropriately (sample size range 52–298). In the adjuvant setting, the same search revealed more studies (maximum sample size of 284) demonstrating more often a benefit from adjuvant use.

**Added value of this study**

The most important finding in this study is that there was no relevant clinical benefit with neoadjuvant therapy despite a sample size 5 times larger than the largest previous study. On the contrary, adjuvant therapy was shown to be associated with improved recurrence-free and overall survival.

**Implications of all the available evidence**

The vast usage of neoadjuvant therapy prior to cytoreductive surgery and HIPEC treatment of colorectal peritoneal metastases (even as part of some countries standard treatment algorithms) is brought into serious questioning. Reassessment of neoadjuvant therapy is warranted pending future randomized trials, while adjuvant therapy continues to hold a potential clinical benefit.

## Introduction

Stage IV colorectal cancer remains an area of concerted efforts to help increase patient survival rates with both medical and surgical strategies being employed to not only increase survival times but also the proportion of patients reaching long-term recurrence-free survival. It is well-known that peritoneal metastases have a worse prognosis than other metastatic sites.^1^ Systemic chemotherapy including biologic therapy for this type of metastatic disease still provides poor results in multicentre randomised trials with a median overall survival of approximately 17 months.^1^ However, cytoreductive surgery (CRS) with or without hyperthermic intraperitoneal chemotherapy (HIPEC) has shown some remarkable results with overall survival reaching >40 months for select patients in a recent randomised controlled trial.^2^ Today, many countries have incorporated CRS/HIPEC into their national guidelines for the treatment of colorectal cancer with peritoneal metastases.^3^

The use of perioperative systemic chemotherapy in the setting of CRS/HIPEC remains a contentious issue. There are no randomised trials to help guide decision making, even though there is one trial currently recruiting – CAIRO 6.^4^^,^^5^ There are several larger case series that have in multivariable analyses shown that adjuvant therapy may have a positive effect and one propensity score matched study.^6^, ^7^, ^8^ On the other hand, several studies have not shown the same positive outcome for adjuvant therapy^7^^,^^9^^,^^10^. The use of neoadjuvant therapy is even more controversial. To date, there are basically no large observational studies supporting the use of neoadjuvant therapy in conjunction with CRS/HIPEC only very small case series.^11^, ^12^, ^13^, ^14^, ^15^, ^16^ Equally, in liver-only metastatic colorectal cancer the utility of adjuvant chemotherapy has increasingly been questioned (JCOG0603 Trial).^17^

At the biennial PSOGI 2018 international conference, a collaborative group was formed to study the effect of various HIPEC regimens. Using this large global cohort, the present study aims to evaluate the efficacy of perioperative systemic chemotherapy in conjunction with CRS/HIPEC for patients with colorectal cancer with peritoneal metastases (CRCPM). The primary endpoints are overall survival and recurrence-free survival. Perioperative systemic chemotherapy in the form of neoadjuvant and adjuvant systemic chemotherapy will be evaluated separately in two propensity score matching analyses.

## Methods

### Study design

The study used the global PSOGI initiated database on CRCPM, which locked its data capture through audits from two separate investigators (PC & OF) in 2020. The registry spans from January 1, 1991, to December 31, 2018, from 39 HIPEC centres globally. Details on data capture and establishing the base cohort of 2093 patients are described in the Supplementary Methods section. Briefly, this PSOGI registry included all patients suffering from CRCPM from each respective centre's HIPEC registries. Only patients having undergone CRS+HIPEC treatment were included in the PSOGI compiled registry. The retrospective capture of data for this study was approved by each centres' national registries' respective ethical review boards. All data was anonymised prior to registry synchronisation so informed consent was not required. Certain variables were deemed completely necessary and not possible to estimate in a reasonable manner. All patients with missing PCI (peritoneal cancer index), CC scores (completeness of cytoreduction), and specified HIPEC treatment drug regimen were removed from the 2093 base cohort. Demographics, clinicopathological variables, operating variables, postoperative morbidity, overall survival, and recurrence-free survival was retrieved from the PSOGI compiled registry.

**Fig. 1:**
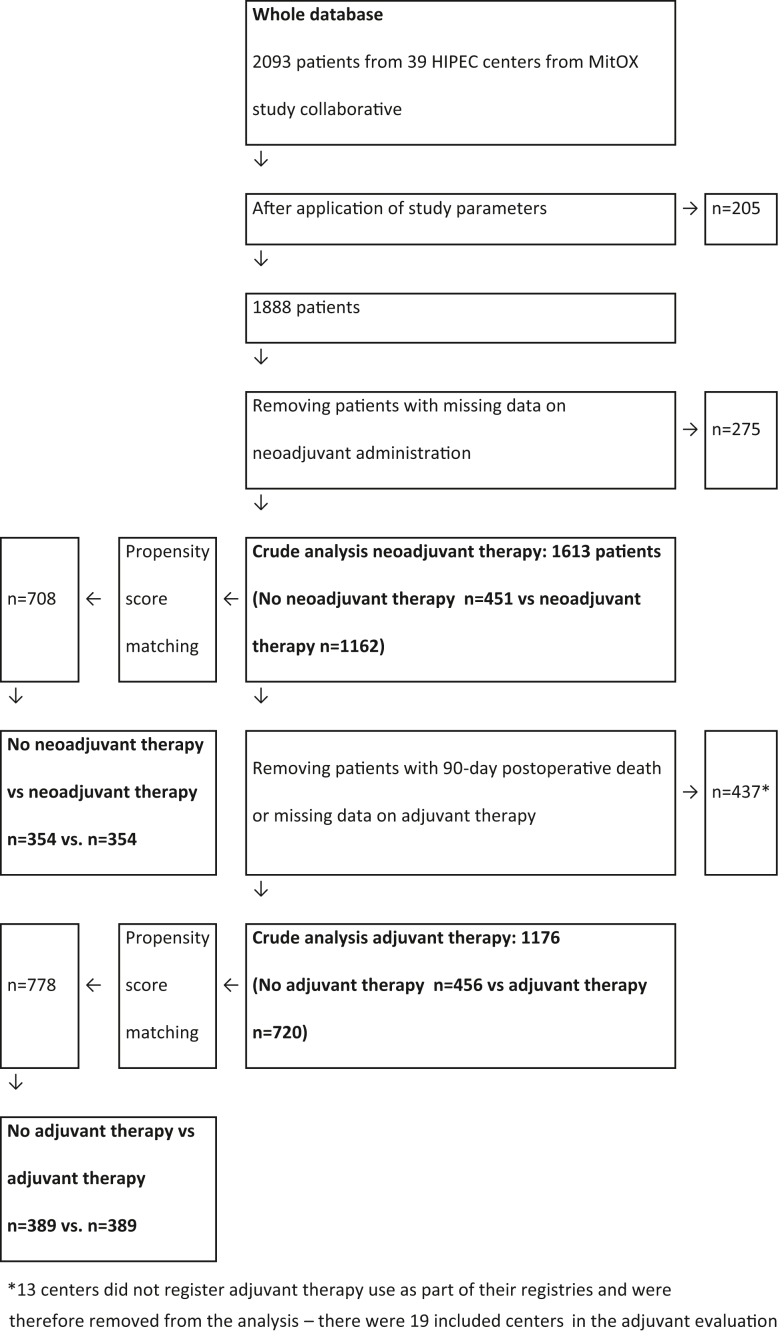
Flowchart of patient data selection & analysis.

This study aimed to evaluate neoadjuvant therapy use and adjuvant therapy use through two separate propensity score matched analyses. For the neoadjuvant therapy evaluation, all patients with missing data on neoadjuvant therapy were removed (Fig. 1). Patients with missing adjuvant therapy data were allowed in the neoadjuvant therapy evaluation. When moving to the adjuvant therapy evaluation, patients with missing data on adjuvant therapy or patients having died within 90 days were removed for this analysis (Fig. 1).

Data on exact systemic chemotherapy drugs used and doses were not available in the registry. As all centres have had different strategies for perioperative systemic chemotherapy use, the study committee opted for a two-step study design. The study aimed to mimic the clinical situation of when treatment decisions are made; first, preoperative multi-disciplinary therapy (MDT) conference decide for neoadjuvant therapy or not; and secondly, the postoperative MDT conference where the decision of whether to administer adjuvant therapy is made. As not all patients received neoadjuvant or adjuvant therapy, a propensity score matching procedure was performed to create comparable groups in both clinical situations. Overall survival follow-up was required for all patients. Follow-up for recurrences and adjuvant therapy was systematically missing from a few centres’ HIPEC registries that did not collect this data. They were allowed to be included in the neoadjuvant therapy evaluation. In the adjuvant therapy evaluation, these centers were removed as missing data on adjuvant therapy was not deemed imputable for this analysis (Fig. 1).

### Neoadjuvant propensity score matching

All patients with data on neoadjuvant therapy use comprised the crude neoadjuvant evaluation cohort. Within this group, a propensity score matching was performed using the clinical factors that are known at the preoperative MDT: sex, age, presence of liver metastases, HIPEC centre, lymph node positive disease, synchronous or metachronous disease, peritoneal cancer index (PCI) as a surrogate marker for preoperative CT-PCI, and the planned HIPEC regimen.

### Adjuvant propensity score matching

From the crude neoadjuvant evaluation cohort, all patients with missing adjuvant therapy data or with postoperative death within 90 days of CRS/HIPEC were removed to define the crude adjuvant evaluation cohort (Fig. 1). Within this adjuvant evaluation cohort, a propensity score matching was performed using the clinical factors that are known at the postop MDT: same factors for neoadjuvant propensity score matching plus completeness of cytoreduction score and Clavien-Dindo grade 3+ morbidity.

### Statistical analysis

Patient demographics for crude neoadjuvant and adjuvant evaluation groups were calculated together with the two propensity score matched groups. The differences in variables between the two groups in each respective propensity score analyses were calculated using standardized mean differences. There was missing data for timing of peritoneal metastases, primary tumor location, Clavien-Dindo morbidity, adjuvant therapy (only in the neoadjuvant evaluation) and return to OR. These were handled by making the missing data group a separate category. Evaluation of the effect of pre- and postoperative chemotherapy was performed using two analyses. First, a Kaplan–Meier curve with log-rank tests between propensity score matched groups were calculated. Secondly, a multivariable Cox-proportional analysis using all preoperative data and operative data, was conducted for both crude cohorts. The adjuvant evaluation cohort included also Clavien-Dindo morbidity since this is known to affect the ability to administer postoperative adjuvant therapy. Supplementary figures on the covariate balance after propensity score matching was evaluated using Love plots. All propensity score matching was performed using R statistical software with the MatchIt package. The nearest neighbor method was used with a caliper of 0.1 for both propensity score matching analyses. Kaplan–Meier curves were rendered, and log-rank tests performed with Statistica v13 software. All data with p-value <0.05 was considered statistically significant. The proportional hazard assumption was tested with the Grambsch–Therneau test.

Using the 10-year follow-up of death events (1399 events) and the 1:2.6 ratio of no neoadjuvant therapy to neoadjuvant therapy with alpha 0.05 and power 0.8, a hazard ratio difference of 0.846 is attainable in the Cox-proportional model for evaluating the benefit of administering neoadjuvant systemic chemotherapy. Likewise using 981 death events and 1:1.6 ratio of no adjuvant therapy to adjuvant therapy with alpha 0.05 and power 0.8, a hazard ratio difference of 0.832 is attainable in the Cox-proportional model for evaluating the benefit of administering adjuvant systemic chemotherapy.

### Role of the funding source

There was no funding source for this study. All authors had full access to all data in the study and were responsible for the decision to submit for publication.

## Results

### Propensity score matching process

**Fig. 2:**
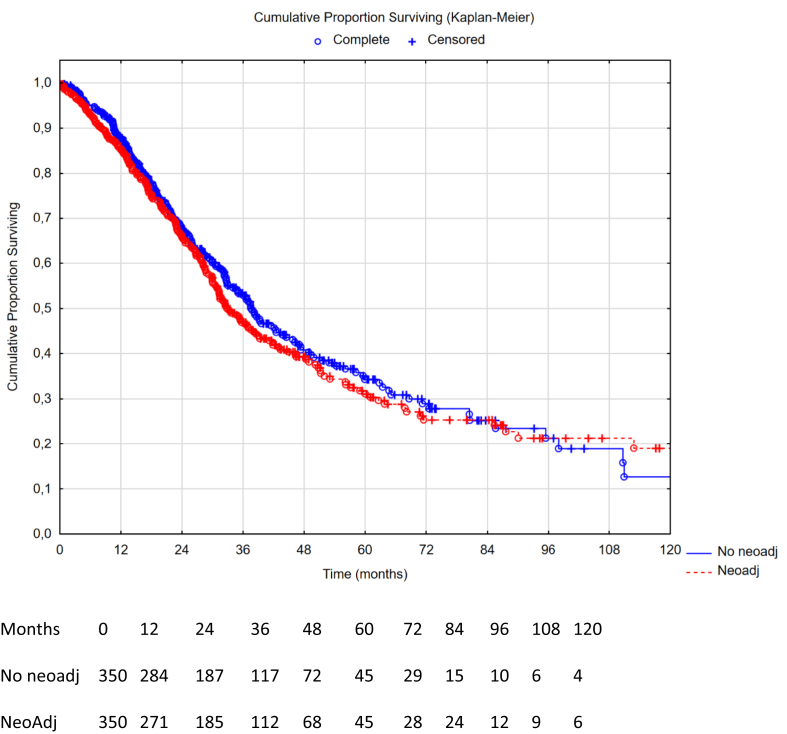
Overall survival comparison between neoadjuvant therapy vs no neoadjuvant therapy in the propensity score matched group (n = 700), missing data on censoring variable or date of death (n = 8). Hazard ratio 1.08 (95% CI: 0.88–1.32), p-value 0.46. Median OS 34.7 months in the neoadjuvant group (95% CI: 31.2–38.8) vs 37.0 months in the no neoadjuvant group (95% CI: 32.6–42.7), log rank p-value 0.46.

**Fig. 3:**
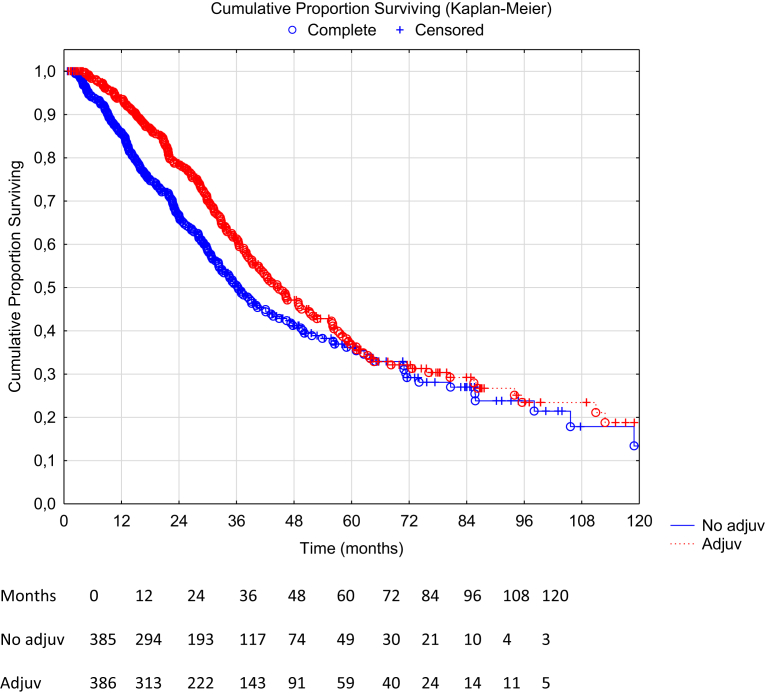
Overall survival comparison between adjuvant therapy vs no adjuvant therapy in the propensity score matched group (n = 771), missing data on censoring or date of death (n = 7). Hazard ratio 0.79 (95% CI: 0.64–0.97), p-value 0.022. Median OS 45.7 months in the adjuvant group (95% CI: 38.8–56.2) vs 37.0 months in the no adjuvant group (95% CI: 32.9–41.8), log rank p-value 0.022.

**Fig. 4:**
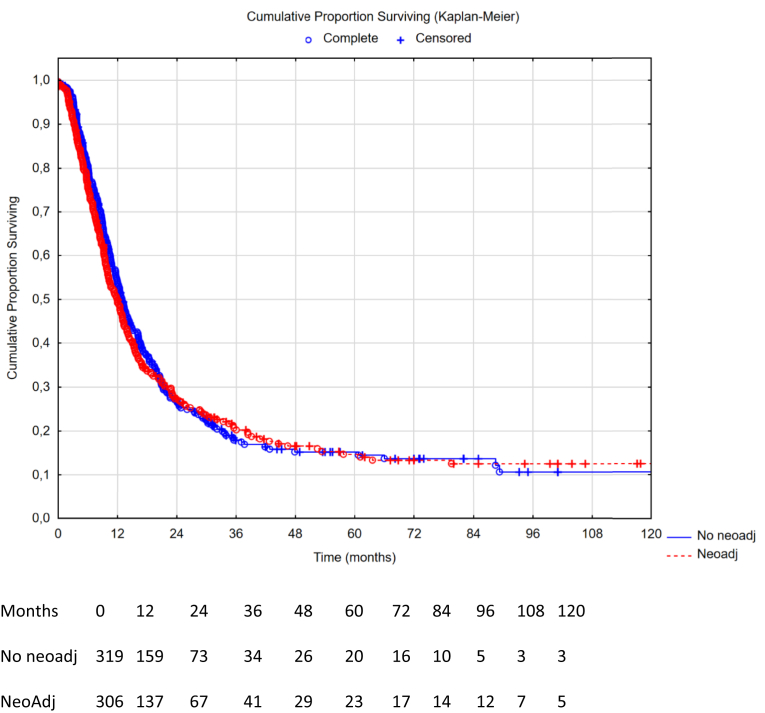
Recurrence-free survival comparison between neoadjuvant therapy vs no neoadjuvant therapy in the propensity score matched group (n = 625), missing data on recurrences (n = 83). Hazard ratio 1.04 (95% CI: 0.87–1.25), p-value 0.66. Median OS 12.3 months in the neoadjuvant group (95% CI: 10.9–13.6) vs 12.6 months in the no neoadjuvant group (95% CI: 11.2–14.1), log rank p-value 0.66.

**Fig. 5:**
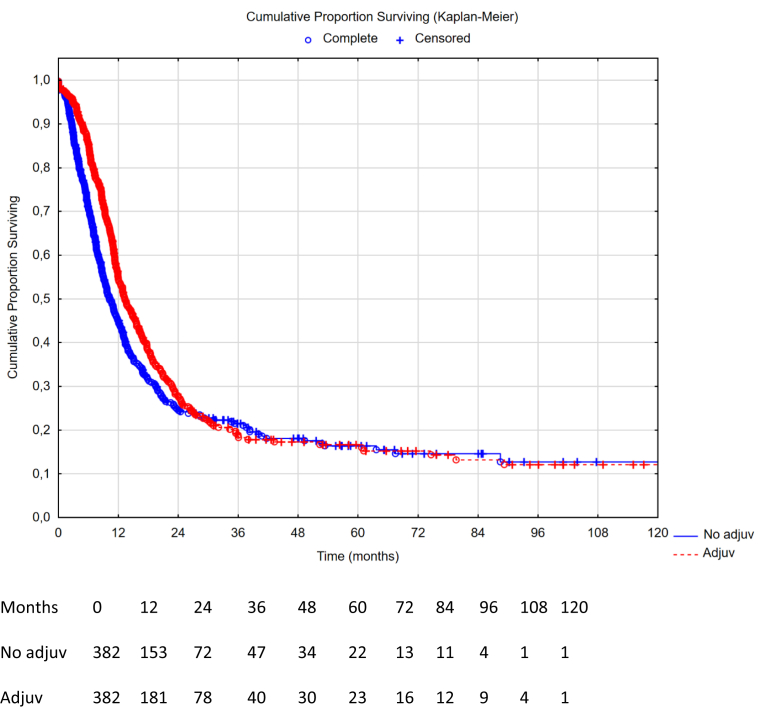
Recurrence-free survival comparison between adjuvant therapy vs no adjuvant therapy in the propensity score matched group (n = 764), missing data on recurrences (n = 14). Hazard ratio 0.83 (95% CI: 0.70–0.98), p-value 0.030. Median RFS 12.7 months in the adjuvant group (95% CI: 11.6–14.7) vs 11.1 months in the no adjuvant group (95% CI: 10.2–12.5), log rank p-value 0.030.

A flowchart of the patient selection process is presented in Fig. 1. The crude neoadjuvant evaluation cohort included 1613 patients and the crude adjuvant evaluation cohort included 1176 patients. The propensity score matched cohort for neoadjuvant evaluation successfully matched 708 patients (354 patients in both the no-neoadjuvant arm and neoadjuvant arm). Likewise, propensity score matching in the adjuvant evaluation resulted in 778 matched patients (389 patients in both the no-adjuvant arm and adjuvant arm). Patient demographics and differences between propensity score cohorts are presented in Table 1. Overall survival (OS) and recurrence-free survival (RFS) times are shown in Fig. 2, Fig. 3, Fig. 4, Fig. 5. Love plots for covariate balance results are shown in Supplemental Figs. S1 and S2. Overall distance reached <0.1 in both propensity score analyses.

**Table 1:** Patient demographics and treatment variables.

Variables	Crude analysis: neoadjuvant n = 1613	PSM: no neoadjuvant n = 354	PSM: neoadjuvant n = 354	SMD	Crude analysis: adjuvant n = 1176	PSM: no adjuvant n = 389	PSM: adjuvant n = 389	SMD
Male sex n (%)	723 (45)	153 (43)	165 (47)	0.068	532 (45)	166 (43)	163 (42)	0.016
Age mean (SD)	56 (12)	56 (13)	55 (12)	0.049	57 (12)	57 (12)	57 (12)	0.010
Liver metastases n (%)	208 (13)	39 (11)	52 (15)	0.110	178 (15)	48 (12)	55 (14)	0.053
Node positive n (%)	1185 (73)	266 (75)	253 (72)	0.083	856 (73)	283 (73)	282 (72)	0.006
Timing of PM n (%)				0.091				0.075
Metachronous	797 (50)	183 (52)	167 (47)		594 (51)	212 (55)	209 (54)	
Synchronous	746 (46)	154 (43)	168 (48)		577 (49)	174 (45)	179 (46)	
Missing data	70 (4)	17 (5)	19 (5)		5 (<1)	3 (<1)	1 (<1)	
Neoadj chemo n (%)	1162 (72)	0 (0)	354 (100)	N/A	891 (76)	285 (73)	285 (73)	0.012
Adjuvant chemo n (%)	739 (46)	66 (19)	74 (21)	0.081	720 (61)	0 (0)	389 (100)	N/A
Missing adjuvant data n (%)	409 (25)	188 (53)	174 (49)		0 (0)	0 (0)	0 (0)	N/A
No periop chemo n (%)	122 (8)	100 (28)	0 (0)	N/A	120 (10)	104 (27)	0 (0)	N/A
PCI mean (SD)	10.1 (7.1)	10.9 (7.3)	11.8 (7.6)	0.114	9.6 (6.9)	9.8 (7.6)	9.5 (6.7)	0.038
CC score 0 n (%)	1498 (93)	330 (93)	316 (89)	0.172	1110 (94)	369 (95)	370 (95)	0.010
CC score 1	85 (5)	20 (6)	26 (7.3)		59 (5)	18 (5)	17 (4)	
CC score 2	21 (1)	3 (<1)	10 (2.8)		5 (<1)	2 (<1)	2 (<1)	
CC score 3	9 (<1)	1 (<1)	2 (0.6)		2 (<1)	0 (0)	0 (0)	
HIPEC n (%)				0.023				0.043
Cisplatin	3 (<1)	1 (<1)	1 (<1)		3 (<1)	1 (<1)	1 (<1)	
Irinotecan	4 (<1)	2 (<1)	2 (<1)		4 (<1)	2 (<1)	1 (<1)	
Mitomycin	659 (41)	155 (44)	151 (43)		400 (34)	146 (38)	144 (37)	
Oxaliplatin ± irinotecan	947 (59)	196 (55)	200 (56)		769 (65)	240 (62)	243 (63)	
Primary tumour n (%)				0.141				0.049
Colon	1322 (82)	285 (80)	293 (83)		1075 (91)	361 (93)	356 (92)	
Rectum	124 (8)	34 (10)	21 (6)		93 (8)	26 (7)	31 (8)	
Missing data	167 (10)	35 (10)	40 (11)		8 (1)	2 (<1)	2 (<1)	
Clavien-Dindo 3+				0.099				0.096
No	897 (56)	207 (59)	216 (61)		714 (61)	221 (57)	239 (61)	
Yes	527 (33)	107 (30)	92 (26)		433 (37)	159 (41)	143 (37)	
Missing data	189 (12)	40 (11)	46 (13)		29 (2)	9 (2)	7 (2)	
Postop mortality n (%)	28 (1.7)	2 (0.6)	6 (1.7)	0.116	N/A	N/A	N/A	0
Return to OR n (%)				0.085				0.039
No	1153 (72)	250 (71)	261 (74)		943 (80)	309 (79)	315 (81)	
Yes	226 (14)	51 (14)	50 (14)		164 (14)	61 (16)	56 (14)	
Missing data	234 (14)	53 (15)	43 (12)		69 (6)	19 (5)	18 (5)	
Median surgery date	18-07-2012	12-12-2011	06-09-2011	0.032	15-06-2013	21-02-2013	08-08-2013	0.161

CC – completeness of cytoreduction, HIPEC – hyperthermnic intraperitoneal chemotherapy, OR – operating room, N/A – not applicable, PCI – peritoneal cancer index, PSM – propensity score matched, SD – standard deviation, SMD – standardized mean differences.

### Survival in the propensity score matched groups

The median OS in the PSM cohort for patients receiving no neoadjuvant vs neoadjuvant therapy was 37.0 months, 95% CI: 32.6–42.7, vs 34.7 months, 95% CI 31.2–38.8, yielding a HR 1.08, 95% CI: 0.88–1.32, p = 0.46. The median OS for patients in the PSM cohort receiving no adjuvant vs adjuvant therapy was 37.0 months, 95% CI 32.9–41.8, vs 45.7 months, 95% CI: 38.8–56.2, yielding a HR 0.79, 95% CI: 0.64–0.97, p = 0.022. The median RFS in the PSM cohort for patients receiving no neoadjuvant vs neoadjuvant therapy was 12.6 months, 95% CI: 11.2–14.1, vs 12.3 months, 95% CI: 10.9–13.6, yielding a HR 1.04, 95% CI: 0.87–1.25, p = 0.66. The median RFS in the PSM cohort for patients receiving no adjuvant vs adjuvant therapy was 11.1 months, 95% CI: 10.2–12.5, vs 12.7 months, 95% CI: 11.6–14.7, yielding a HR 0.83, 95% CI: 0.70–0.98, p = 0.030.

### Survival and Cox regression of crude cohorts

Median OS in the crude cohort for neoadjuvant evaluation was 39 months (95% CI: 36–43) for patients receiving neoadjuvant therapy compared to 37 months (95% CI: 35–42) for those not receiving neoadjuvant therapy, hazard ratio 0.93 (95% CI: 0.80–1.09), p-value 0.39. Median OS in the crude cohort for adjuvant evaluation was 44 months (95% CI: 39–50) for patients receiving adjuvant therapy compared to 38 months (95% CI: 35–42) for those not receiving adjuvant therapy, hazard ratio 0.83 (95% CI: 0.70–0.99), p-value 0.035. Multivariable Cox-proportional hazard modelling in the crude cohorts showed a hazard ratio 1.08 (95% CI: 0.92–1.26, p = 0.37) for administering neoadjuvant therapy and 0.86 (95% CI: 0.72–1.03, p = 0.095) for administering adjuvant therapy (Table 2). The proportional hazards assumption was not violated in the multivariable analysis in Table 2.

**Table 2:** Multivariable analysis on crude cohort for overall survival – neoadjuvant and adjuvant crude cohorts.

Variables with hazard ratios and 95% confidence intervalls	Crude cohort: neoadjuvant evaluation n = 1613	P value	Crude cohort: adjuvant evaluation n = 1176	P value
Male sex	0.98 (0.86–1.16)	0.89	0.93 (0.79–1.12)	0.46
Age	0.99 (0.99–1.00)	0.70	0.99 (0.99–1.00)	0.18
Liver metastases	1.27 (1.03–1.57)	0.024	1.22 (0.96–1.55)	0.11
Node positive	1.46 (1.23–1.75)	<0.0001	1.26 (1.03–1.53)	0.025
Timing of PM				
Metachronous	1.14 (0.99–1.33)	0.67	1.05 (0.88–1.26)	0.66
Synchronous	Reference		Reference	
Missing data	1.18 (0.78–1.81)	0.62	0.76 (0.14–4.01)	0.73
Neoadj chemo	1.05 (0.90–1.23)	0.53	0.96 (0.79–1.16)	0.66
Adjuvant chemo	N/A	N/A	0.86 (0.72–1.03)	0.095
PCI	1.06 (1.05–1.07)	<0.0001	1.05 (1.04–1.07)	<0.0001
CC score 0	Reference		Reference	
CC score 1	1.54 (0.60–3.96)	0.81	1.74 (1.24–2.44)	0.80
CC score 2	2.15 (1.25–3.70)	0.17	4.45 (1.98–10.2)	<0.0001
CC score 3	1.59 (0.50–5.11)^a^	0.93	N/A	
HIPEC				
Cisplatin	0.36 (0.05–2.61)^a^	0.26	0.56 (0.13–2.46)	0.35
Irinotecan	1.10 (0.15–7.95)^a^	0.79	1.35 (0.17–10.6)	0.71
Mitomycin	1.30 (0.27–6.20)	0.10	2.38 (0.69–8.19)	0.39
Oxaliplatin ± irinotecan	Reference		Reference	
Primary tumour				
Colon	Reference		Reference	
Rectum	1.21 (0.94–1.56)	0.029	1.10 (0.81–1.48)	0.75
Missing data	0.78 (0.59–1.05)	0.031	0.46 (0.00–155)	0.75
Clavien-Dindo 3+				
No	N/A		Reference	
Yes	N/A		1.19 (0.99–1.44)	0.13
Missing data	N/A		0.87 (0.50–1.54)	0.44
Date of Surgery	N/A		0.99 (0.99–1.00)	0.72

CC – completeness of cytoreduction, HIPEC – hyperthermic intraperitoneal chemotherapy, NR – not reported, N/A – not applicable or not applied, PCI – peritoneal cancer index, PM – peritoneal metastases, SD – standard deviation.

a Only few cases included – see Table 1.

### Time difference evaluation

Differences in perioperative chemotherapy administration over time was evaluated and median surgery dates are reported in Table 1. There was no statistical difference in median surgery date for the neoadjuvant therapy evaluation (difference of the median surgery date of 97 days, p = 0.68). However, there was a small difference in the adjuvant therapy evaluation (difference of the median surgery date of 168 days, p = 0.033) with the adjuvant therapy group having received more recent treatment than the no adjuvant group (Table 1). This timing difference was added to the multivariable analysis in Table 2, the hazard ratio for receiving adjuvant therapy was 0.86 (95% CI: 0.71–1.02, p-value 0.089) prior to adding the timing variable and was not changed by adding this variable 0.86 (95% CI: 0.72–1.03, p-value 0.095), see Table 2.

## Discussion

In this retrospective, multi-institutional two-step propensity-score matched cohort study, we found that the administration of neoadjuvant chemotherapy to CRS/HIPEC in patients with CRCPM did not add any OS or RFS benefit. On the other hand, administration of adjuvant chemotherapy to CRS/HIPEC was associated with an improvement in OS and RFS. Both primary (propensity score matched Kaplan–Meier with log rank test) and secondary analysis (multivariable Cox regression on the crude cohort) have accounted for postoperative morbidity statistically and postoperative deaths were excluded. Patients receiving adjuvant treatment had an almost 9-month longer median overall survival compared to those who did not receive adjuvant chemotherapy, but the survival curves converge toward 60 months and thus it appears that adjuvant chemotherapy does not increase the overall cure rate. The same results were seen in the recurrence-free survival with a significantly increased short-term effect, yet convergence in the long-term. Nonetheless, it appears that adjuvant therapy may have a potential benefit worth exploring in a randomised setting.

This study is based on large international registries; and due to its size can provide data for relevant clinical decision-making. A major strength of this multicenter study is the broad global inclusion making generalisation of study results more applicable. Adjuvant trials from the medical oncology community have often required sample sizes in the thousands. This study can provide significant power to evaluate relatively small, but clinically relevant differences. For neoadjuvant therapy evaluation, the sample size could evaluate a hazard ratio benefit of 0.846. Similarly, for adjuvant therapy the hazard ratio attainable was 0.832.

In the Netherlands, the CAIRO 6 trial is accruing patients with a target size of 358, which is an important trial since it is a prospective multicentre randomised controlled trial.^4^^,^^5^ The effect size found in our study with a hazard ratio of 0.79 in the univariate analysis and 0.86 as adjusted effect size is out of reach for the current target size in the CAIRO 6 trial. The sample size calculation in the CAIRO 6 trial used a 3-year OS improvement of 15%; however, in our analysis the 3-year difference was ∼10%. The group not receiving adjuvant therapy still had a large proportion receiving neoadjuvant therapy (73%), so it is not completely comparable to the no chemotherapy arm in the CAIRO 6 trial. Nonetheless, our study results may be of importance for the ongoing CAIRO trial to consider.^5^

Looking at the literature, there are no randomised trials and only one well-designed propensity-score matched study to lean on for the evaluation of adjuvant systemic treatment.^8^ However, this study had a very narrow inclusion of patients with only synchronous CRCPM. It could conclude that adjuvant chemotherapy was superior to active surveillance. No evaluation of neoadjuvant therapy was performed. The propensity score matching resulted in a total study size of 284 patients (matching from a cohort of 393 patients). The drawback in the study was that postoperative morbidity was not included as a matching covariate, which is important as it is a strong indicator of a patient that may not be able to receive adjuvant chemotherapy – as is the case with many other major oncologic resections. However, the authors did adjust for this post hoc leading to an adjusted HR of 0.71 (95% CI 0.53–0.95). Other studies on adjuvant therapy have shown mixed results as evidenced in a recent systematic review.^11^ The conclusion from this review was that adjuvant therapy appeared to have less potential for efficacy.^7^^,^^9^^,^^10^ On the contrary, the review had a more positive view on neoadjuvant chemotherapy. However, the neoadjuvant studies included in the review were very small ranging from 91 to 166 patients.^12^, ^13^, ^14^, ^15^, ^16^ The smallest comparator arm ranged from 7 to 41 patients in these studies. With such low study numbers, it is difficult to draw firm conclusions. Our study is the first propensity score matched study evaluating the efficacy of neoadjuvant therapy in conjunction with CRS/HIPEC; and despite large sample sizes of 1613 (whole cohort) and 708 (propensity matched), we have not been able to identify any relevant benefit. Together with the recent study on chemotherapy resistance development in conjunction with neoadjuvant therapy prior to CRS/HIPEC,^18^ it may be more relevant to move towards adjuvant therapy use.

As an example, neoadjuvant use in the relatively chemotherapy sensitive ovarian cancer has been recently evaluated in a comprehensive Cochrane review. The authors concluded that despite several large multi-institutional randomised trials (five RCTs to be exact) met inclusion criteria, neoadjuvant therapy did not add benefit in terms of overall or progression-free survival.^19^ Likewise, it is well-known that systemic therapy for colorectal cancer is much less effective on isolated peritoneal metastases compared to liver or lung metastases.^1^ The systemic treatment is probably more important in preventing systemic metastases, something that might be more relevant postoperatively. However, we hope that the currently active CAIRO 6 trial will provide some more insight into the use of neoadjuvant therapy.^5^

This study has some limitations. Most notably is the lack of systemic chemotherapy details. The use of neoadjuvant or adjuvant therapy can mean a whole range of different therapies of which we do not possess any clinical information in the registry. In preparation for establishing this retrospective cohort, it became evident that most of the prospective national and local registries did not have clinical information on the exact systemic therapies that were utilized. It is, therefore, out of the scope of this study to be able to collect this information and thus it remains elusive to us, what specific therapies were used. All patients have at least received one cycle of systemic chemotherapy to be registered as having received chemotherapy.

A second limitation is the evaluation of neoadjuvant therapy in a retrospective cohort. In a prospective trial, patients receiving neoadjuvant therapy and who progress on it would still be included in the neoadjuvant arm. These patients are not possible to find retrospectively. This drawback probably means that the neoadjuvant group has a better survival than it otherwise would have had, thus not changing the conclusions of this study, rather strengthening them. Furthermore, selection bias is a major concern in the neoadjuvant setting. It could be that patients not receiving chemotherapy are in worse general condition, thus skewing the results in favor of the chemotherapy group. Even with a good propensity score matching, you cannot fully account for selection bias. Nonetheless, despite the probability that the neoadjuvant group is in better shape, this has not led to any visible benefit. Furthermore, we know from the Nordic Peritoneal Oncology Group that most patients from the Nordic centres do not receive neoadjuvant therapy, which is only used selectively in down-staging attempts. This differs significantly from the French centres where neoadjuvant therapy is part of the standard treatment. To account for these differences, matching was performed including HIPEC centres as a matching variable. This also alleviated the centre-related missing data in Fig. 4, where there is a significant number of patients with missing data on recurrence-free survival (some centre registries have not registered RFS). Lastly, neoadjuvant therapy is connected to down-staging use as well. Patients who are initially large-volume and respond to chemotherapy may still have a worse prognostic outcome. However, we believe that matching according to PCI levels alleviates this issue, as PCI is one of the most important prognostic indicators for survival. It is not clear which patient is in a better position, the one who has a PCI 10 during up front surgery or the patient with PCI 10 after responding with regression on neoadjuvant therapy. All things considered; neoadjuvant therapy has not been able to prove itself as having a promising benefit.

Limitations with the adjuvant therapy evaluation exist as well. As was the case with neoadjuvant therapy, the indications for adjuvant use differ with the centres. In many Nordic countries, patients with metachronous PM do not receive adjuvant therapy, particularly if they have already received it after primary tumor resection. Likewise, some patients opt out of adjuvant therapy administration; thus, patients not receiving adjuvant therapy may have differing reasons for this. To account for these differences, the propensity score matching included among other variables, the HIPEC centre, metachronous vs synchronous PM, and Clavien-Dindo 3–5 morbidity. All patients with 90-day mortality were removed prior to matching. We believe that with this matching, the baseline characteristics are very similar between the groups, even though all potential selection biases cannot be ruled out in a retrospective setting. The last limitation is the difference in treatment over time. There was no statistical difference between groups in the neoadjuvant evaluation; however, in the adjuvant evaluation, the patients that received adjuvant therapy got it more recently than the patients not receiving adjuvant therapy. This variable was therefore added to the multivariable analysis in Table 2. This difference in timing did not affect the hazard ratio. However, in the crude multi-variable analysis (Table 2), the p-value does not quite reach 0.05. Thus, the results should be interpreted with some caution.

In conclusion, neoadjuvant therapy did not add any benefit while adjuvant therapy was associated with a benefit that should be verified in a randomised trial.

## Contributors

O.M.F. and P.H.C. were responsible for the final data capture and verification, synchronisation of registry data, study design, statistical analyses, interpretation of study results, first writing draft, revising draft, final approval of revised version, and accountable for all aspects of the work.

J.E., S.G.L., W.L., N.A.A., D.L.M., V.K., I.S., F.D., J.-J.T., C.C., B.D., O.S., F.Q., O.G. provided study registry data, interpreted study results, revised the first draft manuscript, final approval of revised version, and accountable for all aspects of the work. All authors had full access to all data in the study. No funding source was used.

## Data sharing statement

All raw data is available for review upon request. The data will be untraceable to any individual by removing all dates and centre information.

## Declaration of interests

Oliver M. Fisher reports personal fees from GORE and Fisher & Paykel Healthcare outside the submitted work. Olivier Glehen is consultant for GAMIDA.

## Appendix A Supplementary data

Appendix

STROBE checklist

Table Contributors
