# Supplementary material for: Perioperative chemotherapy in colorectal cancer with peritoneal metastases: A global propensity score matched study
Source: eClinicalMedicine. 2022 Nov 24;55:101746. doi: 10.1016/j.eclinm.2022.101746 (PMC9706515; doi:10.1016/j.eclinm.2022.101746)
Supplement: Appendix [file mmc1.pdf]

Captions for supplementary material

Supplementary Figure S1. Covariate balance diagnostics (love plot) – Neoadjuvant propensity score matching process.

Supplementary Figure S2. Covariate balance diagnostics (love plot) – Adjuvant propensity score matching process.

Supplementary Figure S1.

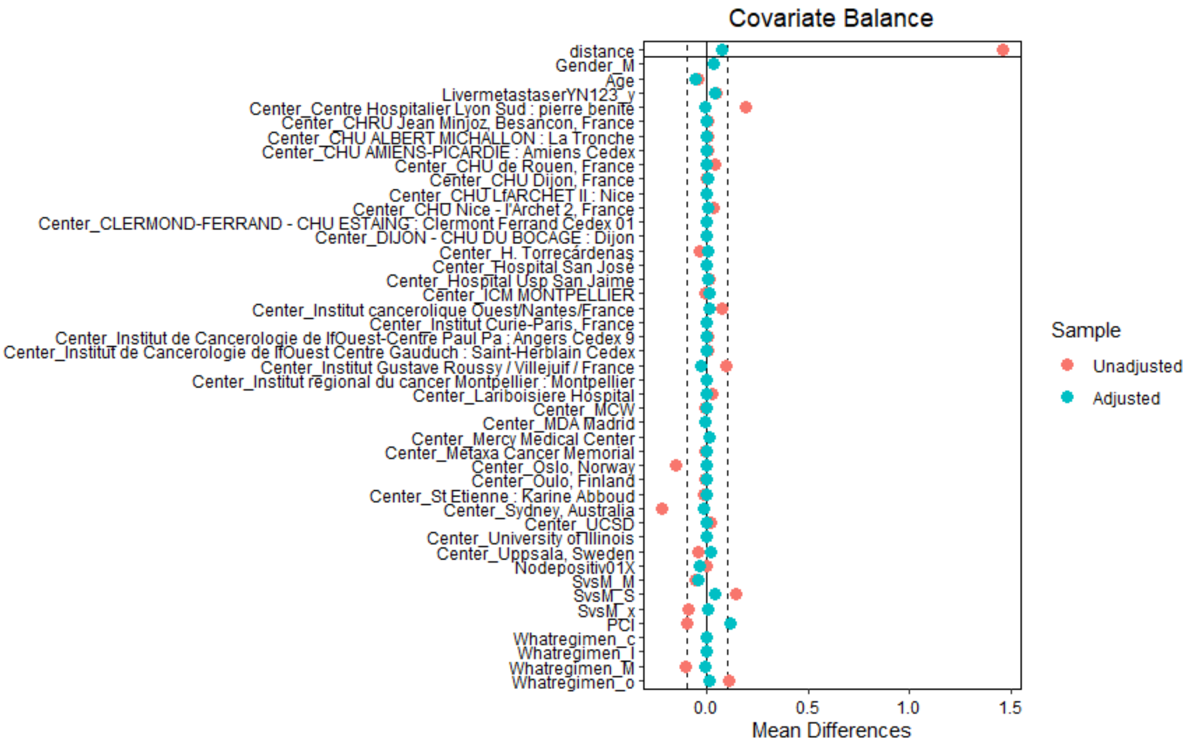

Supplementary Figure S2.

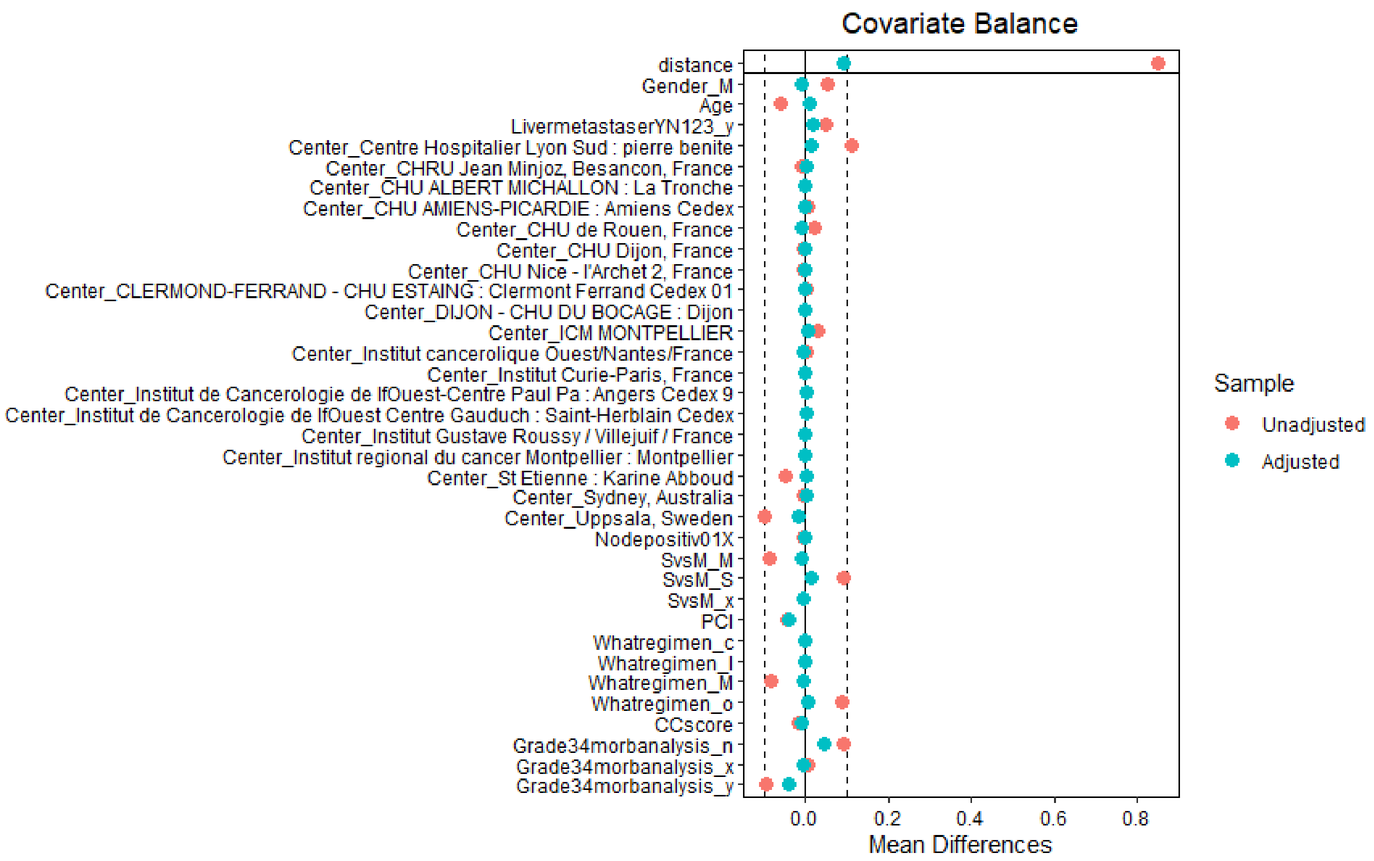

Supplementary methods – data capture

Databases & study population

The MitOxHIPEC study (Mitomycin C vs. Oxaliplatin Hyperthermic Intraperitoneal Chemotherapy combined with Cytoreductive Surgery for the Treatment of Colorectal Cancer with Peritoneal Metastases) was a retrospective, multi-institutional cohort study of clinical and surgical practices regarding the management of patients who underwent CRS/HIPEC for the treatment of CRC with peritoneal metastases. Data were compiled across eight countries and 39 treatment centers that were part of the Peritoneal Surface Oncology Group International (PSOGI), Nordic Peritoneal Oncology Group (NPOG), American Society for Peritoneal Surface Malignancy (ASPSM) and BIG-RENAPE Groups. Data were identified from the prospectively maintained databases of the collaborative groups and compiled in a central, standardized, de-identified and password protected database. During inputting of data into the central database, data were checked for accuracy and completeness with potential duplicate values removed by cross-referencing patients by country and treatment center, patient age, gender, date of CRS and corresponding intraoperative PCIs. Attempts to rectify missing or inconsistent data were obtained from e-mail exchanges with referral centers where possible. To ensure concordance between databases and data integrity, final data capture was audited by two of the investigators (OMF & PC) prior to analysis.

First Name	Last Name	Network collaboration
Kjersti	Flatmark	Nordic Peritoneal Oncology Group (NPOG)
Wilhelm	Graf	Nordic Peritoneal Oncology Group (NPOG)
Heikki	Takala	Nordic Peritoneal Oncology Group (NPOG)
Andrew	Lowy	American Society for Peritoneal Surface Malignancy (ASPSM)
Terence	Chua	American Society for Peritoneal Surface Malignancy (ASPSM)
Joerg	Pelz	American Society for Peritoneal Surface Malignancy (ASPSM)
Dario	Baratti	American Society for Peritoneal Surface Malignancy (ASPSM)
Joel	Baumgartner	American Society for Peritoneal Surface Malignancy (ASPSM)
Richard	Berri	American Society for Peritoneal Surface Malignancy (ASPSM)
Pedro	Bretcha-Boix	American Society for Peritoneal Surface Malignancy (ASPSM)
Marcello	Deraco	American Society for Peritoneal Surface Malignancy (ASPSM)
Guillermo	Flores-Ayala	American Society for Peritoneal Surface Malignancy (ASPSM)
Alberto	Gomez-Portilla	American Society for Peritoneal Surface Malignancy (ASPSM)
Santiago	González-Moreno	American Society for Peritoneal Surface Malignancy (ASPSM)
Martin	Goodman	American Society for Peritoneal Surface Malignancy (ASPSM)
Evgenia	Halkia	American Society for Peritoneal Surface Malignancy (ASPSM)
Shigeki	Kusamura	American Society for Peritoneal Surface Malignancy (ASPSM)
Mecker	Moller	American Society for Peritoneal Surface Malignancy (ASPSM)
Guillaume	Passot	American Society for Peritoneal Surface Malignancy (ASPSM)
Marc	Pocard	American Society for Peritoneal Surface Malignancy (ASPSM)
George	Salti	American Society for Peritoneal Surface Malignancy (ASPSM)
Armando	Sardi	American Society for Peritoneal Surface Malignancy (ASPSM)
Maheswari	Senthil	American Society for Peritoneal Surface Malignancy (ASPSM)
John	Spilioitis	American Society for Peritoneal Surface Malignancy (ASPSM)
Juan	Torres-Melero	American Society for Peritoneal Surface Malignancy (ASPSM)
Kiran	Turaga	American Society for Peritoneal Surface Malignancy (ASPSM)
Jean-Marc	Bereder	BIG-RENAPE
Jean-Louis	Bernard	BIG-RENAPE
Naoual	Bakrin	BIG-RENAPE
Sébastien	Carrère	BIG-RENAPE
Julien	Coget	BIG-RENAPE
Eddy	Cotte	BIG-RENAPE
Olivier	Facy	BIG-RENAPE
Maximiliano	Gelli	BIG-RENAPE
François-Noël	Gilly	BIG-RENAPE
Pablo	Ortega-Deballon	BIG-RENAPE
Guillaume	Passot	BIG-RENAPE
Patrick	Rat	BIG-RENAPE
Pascal	Rousset	BIG-RENAPE
Emilie	Thibaudeau	BIG-RENAPE
Delphine	Vaudoyer	BIG-RENAPE
