# Supplementary material for: Perioperative chemotherapy in colorectal cancer with peritoneal metastases: A global propensity score matched study
Source: eClinicalMedicine. 2022 Nov 24;55:101746. doi: 10.1016/j.eclinm.2022.101746 (PMC9706515; doi:10.1016/j.eclinm.2022.101746)
Supplement: STROBE checklist [file mmc2.pdf]

STROBE Statement—Checklist of items that should be included in reports of ***case-control studies***

	Item No	Recommendation	Pages and lines according to clean version of manuscript
**Title and abstract**	1	(*a*) Indicate the study's design with a commonly used term in the title or the abstract	pg 1
		(*b*) Provide in the abstract an informative and balanced summary of what was done and what was found	pg 4
**Introduction**			
Background/rationale	2	Explain the scientific background and rationale for the investigation being reported	pg 5
Objectives	3	State specific objectives, including any prespecified hypotheses	pg 5, last prgph
**Methods**			
Study design	4	Present key elements of study design early in the paper	pg 6-7
Setting	5	Describe the setting, locations, and relevant dates, including periods of recruitment, exposure, follow-up, and data collection	pg 6, line,5-7, line 8-9; pg 7, first paragr
Participants	6	(*a*) Give the eligibility criteria, and the sources and methods of case ascertainment and control selection. Give the rationale for the choice of cases and controls	pg 6, line 5-10
		(*b*) For matched studies, give matching criteria and the number of controls per case	pg 7, 2-3 paragraph
Variables	7	Clearly define all outcomes, exposures, predictors, potential confounders, and effect modifiers. Give diagnostic criteria, if applicable	pg 5, last line, second paragraph
Data sources/ measurement	8*	For each variable of interest, give sources of data and details of methods of assessment (measurement). Describe comparability of assessment methods if there is more than one group	pg 6, pg 7 first paragraph
Bias	9	Describe any efforts to address potential sources of bias	pg 7 PSM matching, pg 8 multivar.
Study size	10	Explain how the study size was arrived at	pg 8, last 3-4 sentences
Quantitative variables	11	Explain how quantitative variables were handled in the analyses. If applicable, describe which groupings were chosen and why	pg 8, statistics
Statistical methods	12	(*a*) Describe all statistical methods, including those used to control for confounding	pg 8, stats
		(*b*) Describe any methods used to examine subgroups and interactions	pg 8, stats
		(*c*) Explain how missing data were addressed	pg 8, line 2-4
		(*d*) If applicable, explain how matching of cases and controls was addressed	pg 8, stats
		(*e*) Describe any sensitivity analyses	pg 8 stats, multivar analys
**Results**			
Participants	13*	(a) Report numbers of individuals at each stage of study—eg numbers potentially eligible, examined for eligibility, confirmed eligible, included in the study, completing follow-up, and analysed	Figure 1
		(b) Give reasons for non-participation at each stage	Figure 1
		(c) Consider use of a flow diagram	Figure 1
Descriptive data	14*	(a) Give characteristics of study participants (eg demographic, clinical, social) and information on exposures and potential confounders	Table 1
		(b) Indicate number of participants with missing data for each variable of interest	Table 1
Outcome data	15*	Report numbers in each exposure category, or summary measures of exposure	Table 1
Main results	16	(*a*) Give unadjusted estimates and, if applicable, confounder-adjusted estimates and their precision (eg, 95% confidence interval). Make clear which confounders were adjusted for and why they were included	pg 9, 2nd para Table 2
		(*b*) Report category boundaries when continuous variables were categorized	Table 1
		(*c*) If relevant, consider translating estimates of relative risk into absolute risk for a meaningful time period	No use of relative risk

Other analyses	17	Report other analyses done—eg analyses of subgroups and interactions, and sensitivity analyses	Multivariable analyses as sensitivity analyses was performed
**Discussion**			
Key results	18	Summarise key results with reference to study objectives	pg 11, 1st paragraph
Limitations	19	Discuss limitations of the study, taking into account sources of potential bias or imprecision. Discuss both direction and magnitude of any potential bias	pg 13-14
Interpretation	20	Give a cautious overall interpretation of results considering objectives, limitations, multiplicity of analyses, results from similar studies, and other relevant evidence	pg 15
Generalisability	21	Discuss the generalisability (external validity) of the study results	pg 11, 2nd paragraph
**Other information**			
Funding	22	Give the source of funding and the role of the funders for the present study and, if applicable, for the original study on which the present article is based	pg 15, last paragraph

*Give information separately for cases and controls.

Note: An Explanation and Elaboration article discusses each checklist item and gives methodological background and published examples of transparent reporting. The STROBE checklist is best used in conjunction with this article (freely available on the Web sites of PLoS Medicine at http://www.plosmedicine.org/, Annals of Internal Medicine at http://www.annals.org/, and Epidemiology at http://www.epidem.com/). Information on the STROBE Initiative is available at http://www.strobe-statement.org.
