# Supplementary material for: Perioperative chemotherapy in colorectal cancer with peritoneal metastases: A global propensity score matched study
Source: eClinicalMedicine. 2022 Nov 24;55:101746. doi: 10.1016/j.eclinm.2022.101746 (PMC9706515; doi:10.1016/j.eclinm.2022.101746)
Supplement: Table Contributors [file mmc3.docx]

| First Name | Last Name | Network collaboration |
| --- | --- | --- |
| Kjersti | Flatmark | Nordic Peritoneal Oncology Group (NPOG) |
| Wilhelm | Graf | Nordic Peritoneal Oncology Group (NPOG) |
| Heikki | Takala | Nordic Peritoneal Oncology Group (NPOG) |
| Andrew | Lowy | American Society for Peritoneal Surface Malignancy (ASPSM) |
| Terence | Chua | American Society for Peritoneal Surface Malignancy (ASPSM) |
| Joerg | Pelz | American Society for Peritoneal Surface Malignancy (ASPSM) |
| Dario | Baratti | American Society for Peritoneal Surface Malignancy (ASPSM) |
| Joel | Baumgartner | American Society for Peritoneal Surface Malignancy (ASPSM) |
| Richard | Berri | American Society for Peritoneal Surface Malignancy (ASPSM) |
| Pedro | Bretcha-Boix | American Society for Peritoneal Surface Malignancy (ASPSM) |
| Marcello | Deraco | American Society for Peritoneal Surface Malignancy (ASPSM) |
| Guillermo | Flores-Ayala | American Society for Peritoneal Surface Malignancy (ASPSM) |
| Alberto | Gomez-Portilla | American Society for Peritoneal Surface Malignancy (ASPSM) |
| Santiago | González-Moreno | American Society for Peritoneal Surface Malignancy (ASPSM) |
| Martin | Goodman | American Society for Peritoneal Surface Malignancy (ASPSM) |
| Evgenia | Halkia | American Society for Peritoneal Surface Malignancy (ASPSM) |
| Shigeki | Kusamura | American Society for Peritoneal Surface Malignancy (ASPSM) |
| Mecker | Moller | American Society for Peritoneal Surface Malignancy (ASPSM) |
| Guillaume | Passot | American Society for Peritoneal Surface Malignancy (ASPSM) |
| Marc | Pocard | American Society for Peritoneal Surface Malignancy (ASPSM) |
| George | Salti | American Society for Peritoneal Surface Malignancy (ASPSM) |
| Armando | Sardi | American Society for Peritoneal Surface Malignancy (ASPSM) |
| Maheswari | Senthil | American Society for Peritoneal Surface Malignancy (ASPSM) |
| John | Spilioitis | American Society for Peritoneal Surface Malignancy (ASPSM) |
| Juan | Torres-Melero | American Society for Peritoneal Surface Malignancy (ASPSM) |
| Kiran | Turaga | American Society for Peritoneal Surface Malignancy (ASPSM) |
| Jean-Marc | Bereder | BIG-RENAPE |
| Jean-Louis | Bernard | BIG-RENAPE |
| Naoual | Bakrin | BIG-RENAPE |
| Sébastien | Carrère | BIG-RENAPE |
| Julien | Coget | BIG-RENAPE |
| Eddy | Cotte | BIG-RENAPE |
| Olivier | Facy | BIG-RENAPE |
| Maximiliano | Gelli | BIG-RENAPE |
| François-Noël | Gilly | BIG-RENAPE |
| Pablo | Ortega-Deballon | BIG-RENAPE |
| Guillaume | Passot | BIG-RENAPE |
| Patrick | Rat | BIG-RENAPE |
| Pascal | Rousset | BIG-RENAPE |
| Emilie | Thibaudeau | BIG-RENAPE |
| Delphine | Vaudoyer | BIG-RENAPE |
